# Impact of Thermal Cycling on Volumetric Stability of Endodontic Filling Materials

**DOI:** 10.1002/cre2.70400

**Published:** 2026-07-08

**Authors:** Petra Dijanic, Marko Katic, Ivan Tomasic, Ana Ivanisevic, Jurica Matijevic

**Affiliations:** ^1^ School of Dental Medicine University of Zagreb Zagreb Croatia; ^2^ Department of Quality, Faculty of Mechanical Engineering and Naval Architecture University of Zagreb Zagreb Croatia; ^3^ Department of Computer Science and Engineering, Faculty of Engineering and Health Sciences Mälardalen University Västerås and Eskilstuna Sweden; ^4^ Department of Endodontics and Restorative Dental Medicine, School of Dental Medicine University of Zagreb Zagreb Croatia

**Keywords:** endodontic sealers, micro‐CT, obturation, thermal cycling, volume changes

## Abstract

**Objective:**

To evaluate the dimensional stability of root canal obturation materials under dynamic conditions by simulating ageing.

**Materials and Methods:**

Forty extracted, single‐rooted, human teeth were decoronated and randomly assigned to four experimental groups of 10 teeth each. Root canals were instrumented using the Reciproc R40 system and obturated using the single‐cone technique in combination with one of four sealers: AH Plus, MTA Fillapex, TotalFill BC Sealer, or EndoREZ. All specimens underwent thermal cycling (9000 cycles between 5°C and 55°C) to simulate ageing. Each tooth was scanned using high‐resolution micro‐computed tomography (micro‐CT) before and after thermal cycling. Volumetric changes of the filling materials were compared by analyzing the pre‐ and post‐treatment scans in the apical 1 and 2‐mm, and over the total canal volume.

**Results:**

At the apical 1‐mm region, AH Plus exhibited the greatest expansion ($0.016\;{\mathrm{mm}}^{3}$). EndoREZ, MTA Fillapex, and TotalFill BC Sealer showed significantly lower expansion ($0.008,0.003,0.001\;{\mathrm{mm}}^{3}$) than AH Plus. In the apical 2‐mm region, only TotalFill BC Sealer expanded significantly less than AH Plus, with a difference of 0.03 mm^3^.

**Conclusion:**

Thermal cycling induced measurable expansion in all tested endodontic sealers, with the most pronounced effects observed in the apical region. AH Plus exhibited significantly greater expansion than the other materials. Controlled apical expansion may be clinically beneficial by improving sealer adaptation to dentinal walls and enhancing apical sealing without generating detrimental radial stresses. These findings underscore the clinical relevance of long‐term volumetric stability under thermally dynamic conditions and suggest that sealer selection may influence the durability of the apical seal.

## 1 Introduction

Endodontic treatment failure persists despite the development of new sealers, epoxy and bioactive, and methods that are faster and more reliable. Failures are often attributed to inadequate endodontic obturation, characterized by marginal gaps that allow bacterial leakage and subsequent microbial activity (Siqueira 2001; Muliyar et al. 2014).

Gutta‐percha is universally used in root canal obturation (Vishwanath and Rao 2019). The composition of gutta‐percha cones is nominally 75% zinc oxide, with 20% gutta‐percha, with minor amounts of binders, opacifiers, and color pigments (Flake and Johanson 2020). The transformation temperatures of gutta‐percha from the β‐form to the α‐form and from the α‐form to the amorphous phase range between 42°C–49°C and 53°C–59°C, respectively. Volume changes accompany these phase changes. Gutta percha expands slightly during heating and shrinks upon cooling (Lee et al. 1997), so thermocycling may result in voids, which compromise the quality of the canal filling (Wang et al. 2025).

Gutta‐percha is insufficient to achieve an effective seal of the root canal system (Flake and Johanson 2020; Mishra et al. 2020); the root canal sealer is a critical component in achieving an effective seal. However, as highlighted in the literature, none of the currently available sealers possess all the ideal characteristics described by Grossman (Aminoshariae et al. 2022; Flake and Johanson 2020). Previous studies have shown that neither the obturation technique nor the sealer mixing method can ensure a completely void‐free filling of the endodontic space (Başer Can et al. 2017; Celikten et al. 2016, 2015; De‐Deus et al. 2015).

Numerous studies have investigated the quality of obturation of root canal fillings, investigated with destructive techniques (dye penetration, glucose penetration, bacterial leakage) (Ballullaya 2017; Karapınar‐Kazandağ et al. 2010; Teoh et al. 2017; Yanpiset et al. 2018). Fewer studies have used micro‐CT to evaluate sealing (Celikten et al. 2015; Huang et al. 2017; Viapiana et al. 2016; Haridas et al. 2024; Komabayashi et al. 2020). Significant effects of thermomechanical fatigue testing (combined cyclic mechanical loading and thermal cycling) were reported for the coronal microleakage of adhesives placed over obturated root canals and on the push‐out bond strength of different root canal sealers (Ebert et al. 2009; Smran et al. 2023; Almousa et al. 2025); however, little data exists on the behavior of root canal filling materials under thermal cycling. This gap in the literature highlights the novelty of the present study, thermal cycling with advanced imaging to simulate long‐term intraoral conditions. Thermal cycling is a widely used test method to simulate the physiological aging of dental restoratives. A protocol of 9000 cycles over a temperature range of 5°C–55°C approximates about 1 year of functional intra‐oral service (Gale and Darvell 1999).

This study aimed to measure dimensional stability using high‐resolution micro‐CT analysis after thermocycling of root canal obturation materials. This research combines three key elements relevant for clinical practice: (1) inclusion of four types of frequently used sealer formulations—epoxy resin‐based (AH Plus), methacrylate resin‐based (EndoREZ), resin‐based MTA Fillapex, and bioactive cement‐based (TotalFill BC Sealer); (2) simulation of long‐term clinical aging through accelerated and amplified thermal cycling (9000 cycles between 5°C and 55°C) and (3) three‐dimensional, non‐destructive assessment of sealer volume over time using micro‐CT. This approach allows for a more comprehensive understanding of how different sealers perform under dynamic intraoral conditions.

The null hypothesis was that no dimensional changes would occur in the obturation materials as a result of thermal cycling.

## 2 Materials and Methods

This manuscript conforms to Preferred Reporting Items for Laboratory studies in Endodontology (PRILE) 2021 guidelines (Nagendrababu et al. 2021a, 2021b). Figure S1 shows the PRILE flowchart.

The research was approved by the Ethical Committee of the School of Dental Medicine, University of Zagreb (Ethical approval No. 05‐PA‐15‐10/2017).

### 2.1 Experimental Procedure

#### 2.1.1 Sample Selection and Tooth Preparation

The study was conducted using 40 single‐rooted human teeth, freshly extracted for periodontal, orthodontic, or surgical reasons. Following extraction, the outer surfaces of the teeth were mechanically cleaned using Gracey curettes (Carl Martin, Solingen, Germany) and disinfected with a 0.5% chloramine‐T solution. The teeth were stored in distilled water at room temperature.

Tooth selection was based on radiographic examination and transillumination using a Bluephase polymerization LED lamp (Ivoclar Vivadent, Schaan, Liechtenstein) with an intensity of 1200 mW/cm^2^. Labiolingual and mesiodistal radiographs were used to exclude teeth with incomplete root formation, more than one root canal, previous endodontic treatment, or teeth with fractures or cracks. The teeth were decoronated using a low‐speed diamond saw (Isomet 1000, Buehler, Illinois, USA) under water cooling, standardizing the root length to 13 mm. The teeth were randomly allocated to four groups (10 per group) using random numbers generated by RANDOM.ORG.

#### 2.1.2 Working Length Determination

The coronal third of each root canal was widened using a Gates‐Glidden drill #3 (Dentsply Maillefer, Ballaigues, Switzerland). A size K‐file #15 (VDW, Munich, Germany) was inserted into the canal until the tip of the instrument was visible at the apical foramen. The working length was established by retracting the instrument 1 mm.

#### 2.1.3 Mechanical and Chemical Root Canal Treatment

Root canals were irrigated with 2.5% sodium hypochlorite, and mechanical instrumentation was performed using the engine‐driven Reciproc system (VDW, Munich, Germany) according to the manufacturer's instructions. A Reciproc R40 instrument was used, powered by a VDW Silver Motor (VDW, Munich, Germany) in the “RECIPROC ALL” mode. The instrument was advanced into the canal using a pecking in‐and‐out motion with minimal force and an amplitude of no more than 3 mm. Initially, instrumentation was performed up to two‐thirds of the working length, and then to the full working length.

After three insertions of each instrument, it was removed, cleaned, and canal patency was verified with a #15 ISO K‐file. Irrigation was performed between each insertion using a 2.5% sodium hypochlorite solution (Zagreb City Pharmacy, Galenski Laboratory, Zagreb, Croatia) with a 2 mL BD Discardit II syringe (Becton Dickinson, Spain) and a BD Microlance 3 needle (0.5 × 25 mm, 25 G, Becton Dickinson, Spain). A total of 15 mL of 2.5% NaOCl solution was used per canal during instrumentation.

The final irrigation protocol included the sequential use of 2 mL of saline solution (B. Braun Adria d.o.o., Zagreb, Croatia), 17% EDTA solution (Pulpdent Corporation, Watertown, USA) each applied for 1 min, then 2 mL of saline solution, and 2 mL of 2% chlorhexidine solution (Zagreb City Pharmacy, Galenski Laboratory, Zagreb, Croatia). The canals were dried with Reciproc R40 paper points (VDW, Munich, Germany).

#### 2.1.4 Root Canal Filling

Cleaned and shaped canals were filled using the single gutta‐percha cone technique and four widely used sealers: the epoxy‐based AH Plus, the methacrylate‐based EndoREZ, the resin‐based MTA Fillapex, and the bioactive cement‐based TotalFill BC Sealer (Dentsply 2025; Angelus 2025; Ultradent Products Inc. 2025; FKG swiss endo 2025). The chemical compositions of the sealers are listed in Table 1.

**Table 1:** The chemical components of the sealers.

AH plus		EndoREZ		MTA Fillapex		TotalFill BC
Epoxide paste	Amine paste	Catalyst	Base	Paste A	Paste B	Paste
Bisphenol A (25%–50%)	1‐adamantane amine (1%–5%)	Diurethane Dimethacrylate (≥ 1%–< 40%)	Diurethane Dimethacrylate (> 10%–≤ 25%)	Salicylate resin (20%–25%)	Fumed Silica	Tricalcium silicate (20.0%–35.0%)
Bisphenol F (3%–< 10%)	N,N'‐dibenzyl‐5‐oxa‐nonandiamine‐1,9 (7%–13%)	Triethylene Glycol Dimethacrylate (> 10%–≤ 25%)	Triethylene Glycol Dimethacrylate (> 10%–≤ 25%)	Calcium Tungstate (20%–25%)	Titanium Dioxide (1%–10%)	Dicalcium silicate (7.0%–15.0%)
Calcium tungstate	TCD‐Diamine (0.1%–1%)	Trade Secret (1%–10%)	Organophosphine Oxide (≥ 0.1%– < 1%)	Fumed Silica	Mineral Trioxide Aggregate (20%–25%)	Zirconium oxide (35.0%–45.0%)
Zirconium oxide	Calcium tungstate		Benzoyl Peroxide (≥ 0.1%–< 1%)		Base resin (20%–25%)	Calcium hydroxide (1.0%–4.0%)
Fumed silica (≤ 2.5%)	Zirconium oxide		Trade Secret (1%–10%)			Unidentified liquid
Pigment	Fumed silica (≤ 2.5%)		Trade Secret (≥ 1%–< 5%)			
	Silicone oil		Glycerol Dimethacrylate (≥ 1%–< 10%)			

The fit of a Reciproc R40 gutta‐percha cone to the predetermined working length was verified before obturation with one of 4 sealers in the following groups.

Group 1: AH Plus sealer (Dentsply Sirona, Konstanz, Germany)

Group 2: MTA Fillapex (Angelus, Londrina, Brazil)

Group 3: TotalFill BC (FKG Dentaire Sàrl, Le Crêt‐du‐Locle, Switzerland)

Group 4: EndoREZ (Ultradent Products, South Jordan, Utah, USA)

AH Plus and MTA Fillapex sealers were hand‐mixed according to the manufacturers' instructions. EndoREZ was mixed using the manufacturer's automixing tips. TotalFill BC Sealer was used as a premixed, ready‐to‐use sealer supplied in an injectable syringe. The gutta‐percha cone was coated with the sealer and inserted into the root canal. Excess gutta‐percha was removed coronally using a heated endodontic plugger (Hu‐Friedy, Chicago, IL, USA) followed by vertical compaction. Approximately 1 mm of gutta‐percha was removed coronally, after which the cavity was coated with Adhese Universal VivaPen (Ivoclar Vivadent, Schaan, Liechtenstein) and restored with Luminos UN composite material (UnoDent, Witham, United Kingdom). Light‐curing was performed for 40 s using a Bluephase LED polymerization lamp. After filling, the samples were stored at 100% humidity and 37°C for 24 h before creation of a simulated PDL.

#### 2.1.5 Periodontal Ligament (PDL) Simulation

Conical plastic tubes (16 mm in diameter, 20 mm in height) were used as molds to create a simulated PDL. The molds were filled with ProBase Cold auto‐polymerizing acrylic resin (Ivoclar Vivadent, Schaan, Liechtenstein). Each tooth was pressed into the acrylic before complete polymerization, creating simulated alveolar bone. After full setting of the acrylic, the impressions of the teeth were filled with Express XT Light Body silicone (3M ESPE, Neuss, Germany), and an obturated root was inserted before the impression material set; the silicone served as simulated PDL. Each root protruded 4 mm above the acrylic surface. After setting, excess silicone was removed from the blocks' surfaces with a scalpel.

#### 2.1.6 Micro‐CT Scanning

The blocks were scanned using a Nikon XT‐H 225 metrology CT scanner, with a geometric resolution of 49 µm. Following thermocycling, a second micro‐CT scan was performed under the same scanning parameters as the initial scan.

#### 2.1.7 Thermal Cycling

After the initial scan, the acrylic blocks were thermocycled to simulate aging. Each group of blocks was wrapped in cotton gauze and labeled for identification. All four groups were thermocycled simultaneously using a thermocycler apparatus (1100/1200, SD Mechatronik, Feldkirchen‐Westerham, Germany), which was used for 9000 cycles between two water baths (5°C and 55°C), with a dwell time of 10 s in each bath and a transfer time of 10 s.

### 2.2 Micro‐CT Analysis

**Figure 1:**
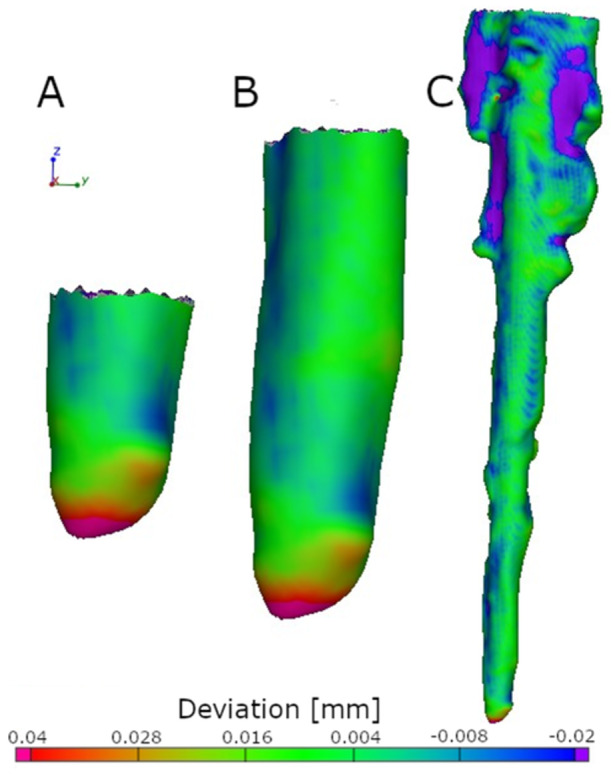
Changes in volume for one tooth (colors represent spatial difference in position of measurement points before and after thermal cycling) (A, 1‐mm apical region; B, 2‐mm apical region; C, total volume).

The before and after thermal cycling micro‐CT scans of each sample were compared using specialized computer software, Volume Graphics Visual Studio Max 2.2. Volumetric changes in the root canal fillings were recorded, and graphical representations were used to visualize areas of specific dimensional changes. The micro‐CT scan of each sample after thermal cycling was superimposed on the scan of the same sample before thermal cycling using an iterative least‐squares optimization algorithm. Then, a built‐in locally adaptive segmentation algorithm was used to differentiate the filling material from the surrounding tooth on both scans. The volume of the filling material after thermal cycling was measured, and the deviation from its pre‐cycling volume was plotted (Figure 1).

### 2.3 Statistical Analysis

The data were exported from the Cone‐Beam Computed Tomography (CBCT) analyzing software and statistically analyzed in R Project for Statistical Computing software (v. 4.4.1). Significance codes used for *p*‐values were 0.001 (***), 0.01 (**), 0.05 (*), and 0.1 (.).

The volumetric changes representing the effects on the sealers were assessed by ANOVA. Pairwise comparisons between sealers were performed using Tukey's post‐hoc test and pairwise *t*‐tests. The assumption of homogeneity of variances was assessed using residuals versus fitted values plots and Levene's test. The assumption of normality was evaluated using a normality plot of residuals and the Shapiro–Wilk test.

Regression modeling was used to correct for the effects of variations in obturation volume on the volumetric change introduced by thermocycling. A linear regression model was fitted for each distance (1‐mm, 2‐mm, and total volume) and allowed to have heteroscedastic variance, that is, different variances for different sealers.

## 3 Results

**Figure 2:**
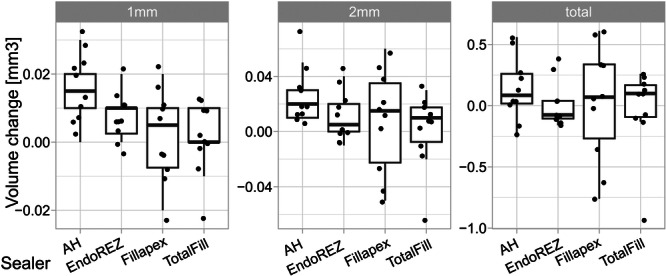
Volumetric changes (volume after—minus before—thermal cycling) for each sample with the median (middle line) and the boxes representing the interquartile range. Note the change in scale from left to right.

An example of dimensional changes measured with micro‐CT is shown in Figure 1. In this tooth, the volumetric increase was largest in the apical region, whereas in the coronal part of the filling, some regions showed contractions. Volumetric changes for all samples in the 1 and 2 mm areas, and the total volume are presented in Figure 2.

### 3.1 ANOVA

ANOVA (Table 2) revealed a statistically significant difference in volumetric changes among the sealers in the 1‐mm apical region (*p* < 0.05). In the 2‐mm apical region and in the total volume, no statistically significant differences were observed among the sealers.

**Table 2:** ANOVA and Tukey multiple pairwise comparisons for different distances (reported are only significant difference).

	Difference $\left[{\mathrm{mm}}^{3}\right]$	*p*
1 mm		0.013*
MTA Fillapex vs. AH Plus	−0.013	0.031*
TotalFill vs. AH Plus	−0.015	0.011*
2 mm	Not significant	
Total	Not significant	

* Significance code: *p *< 0.05.

Tukey's post hoc test in the 1‐mm apical region showed that MTA Fillapex and TotalFill BC Sealer exhibited significantly less expansion compared to AH Plus. The differences in expansion of MTA Fillapex and AH Plus were $-0.013\;{\mathrm{mm}}^{3},\;p=0.03$; the difference for TotalFill BC Sealer and AH Plus was $-0.015\;{\mathrm{mm}}^{3},\;p=0.01$. These findings were confirmed by pairwise *t*‐tests (MTA Fillapex vs AH Plus, *p* = 0.02; TotalFill BC Sealer vs AH Plus, *p* = 0.01). The assumptions were met for homogeneity of variance (Levene's test, *p* > 0.05) and normality of residuals (Shapiro–Wilk test, *p* = 0.567).

### 3.2 Regression Analysis

**Figure 3:**
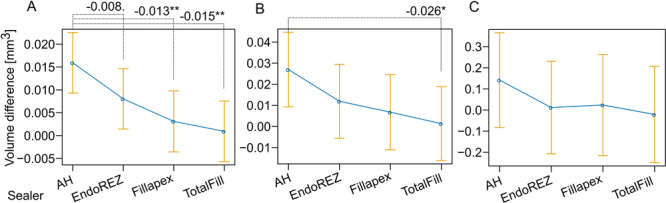
Sealers' effect on volume difference for the 3 distances (A, 1 mm; B, 2 mm; C, total). Whiskers represent the 95% confidence intervals.

Regression analysis (Figure 3) confirmed the ANOVA result that the AH Plus group showed significantly greater expansion than MTA Fillapex and TotalFill BC in the 1‐mm region, while also revealing a significant difference between EndoREZ and AH Plus in the same region (*p* = 0.096). A significant difference was also determined between Totalfill BC Sealer and AH Plus volumes in the 2‐mm region. These additional significant differences were expected because the regression models accounted for root volume and its influence on heat transfer during thermocycling; thicker dentin acts as a thermal insulator to minimize the temperature changes.

Regression analysis was also used to test whether volumetric increases were significant (i.e., significantly different from 0). Table 3 shows the mean increase in volume for each obturation material and each distance. The expansion was significant for AH Plus ($0.016\;{\mathrm{mm}}^{3}$ ***) and EndoRez ($0.008\;{\mathrm{mm}}^{3}$ *) at 1‐mm and for AH Plus at 2‐mm ($0.026\;{\mathrm{mm}}^{3}$ **).

**Table 3:** Increase in volume (${\mathrm{mm}}^{3}$) after thermal cycling for each sealer.

	1‐mm		2‐mm		Total	
	Mean	Sig.	Mean	Sig.	Mean	Sig.
AH	0.016	***	0.026	**	0.141	NS
EndoRez	0.008	*	0.012	NS	0.012	NS
Fillapex	0.003	NS	0.008	NS	0.025	NS
TotalFill	0.001	NS	0.001	NS	−0.022	NS

Abbreviation: NS, non‐significant.

* Significance code: *p* < 0.05.

** Significance code: *p* < 0.01.

*** Significance code: *p* < 0.001.

## 4 Discussion

Micro‐CT analysis enabled precise, three‐dimensional measurements of volume changes before and after thermocycling with a resolution of 49 µm in three regions of the root canal: the apical 1‐ and 2‐mm, and the total volume (Jung et al. 2005; Versiani and Keleș 2020). The data enabled comparisons of the sealers using the same gutta percha. Dimensional stability is an important physicochemical property of root canal sealers with direct clinical relevance. Micro‐CT combined with thermal cycling, provided a non‐destructive method for investigating the dimensional stability of endodontic sealers.

Expansion could be beneficial in a clinical context, as it may enhance the material's adaptation to dentinal walls, reduce interfacial gaps, and improve the seal of the root canal filling. Achieving an effective apical seal is critical to limit apical microleakage. Slight expansion of the sealer may contribute to positive long‐term treatment outcome (Horhat et al. 2023). However, excessive expansion can exert internal hoop stress and predispose the root to fracture. In the present study, the dimensional changes were most pronounced in the apical millimeter.

This study investigated the volume stability of four endodontic sealers (AH Plus, EndoREZ, MTA Fillapex, and TotalFill BC) with a single cone of gutta percha after 9000 thermocycles between 5°C and 55°C. Thermocycling increased the volume of endodontic sealers, especially in the apical region. AH Plus and EndoRez expanded significantly in the apical 1‐mm region, and AH Plus also expanded significantly in the 2‐mm region. Significant differences in volume changes were detected among the tested groups.

The largest volume changes were recorded for the epoxy‐resin sealer AH Plus, whereas EndoREZ, MTA Fillapex, and TotalFill BC Sealer expanded significantly less. A statistically significant difference was observed between AH Plus and TotalFill BC Sealer in the apical 2‐mm region. Since these two materials exhibited significant volumetric change, the null hypothesis was rejected.

The dimensional change of AH Plus may be explained by water sorption after polymerization, because epoxy resin contains hydrophilic monomers. Although epoxy resin‐based sealers are considered dimensionally stable during setting, expansion has been reported by water absorption (Álvarez‐Vásquez et al. 2024; Torres et al. 2019); the present results also indicate significant expansion may occur under thermal cycling.

The mismatch between the coefficient of thermal expansion of the epoxy resin matrix and its inorganic fillers can generate interfacial stress during thermocycling, promoting micro‐void formation at the filler–matrix interface, allowing water sorption, and contributing to hygroscopic swelling and progressive volumetric changes over time. (Álvarez‐Vásquez et al. 2024; Dolgos et al. 2025). Reduced push‐out bond strength of epoxy sealers such as AH Plus after thermocycling supports the concept of interfacial disruption and confirms the formation of micro‐spaces that facilitate fluid ingress. (Almousa et al. 2025). Microvoids would not be detected under the resolution of this micro‐CT (49 µm).

AH Plus exhibits a low film thickness of < 50 µm (Dentsply 2025); however, thicker layers of sealer may accumulate in the apical region of the root canal when used with the single‐cone obturation technique. All sealers tend to pool at the apex; however, if the flow is lower for AH Plus compared to BC sealer (Tosun et al. 2025), more sealer is expected to be present at the apex with single‐cone insertion. MTA Fillapex sealer has a higher flow than AH Plus (Silva et al. 2013). This agrees with Araújo et al.'s results that AH Plus showed a significantly higher sealer volume in the apical 1‐mm compared with Sealapex and Endofill, supporting the role of viscosity in sealer distribution. The presence of a more sealer at the apex may amplify dimensional changes under thermal cycling, contributing to the greater volumetric expansion differences observed in this study.

MTA Fillapex and AH plus are two‐component sealers mixed manually; the two components of EndoRez are mixed via automix syringe system, minimizing air inclusion. Manual mixing of two‐component systems can introduce microporosities that trap air (De‐Deus et al. 2015). When immersed in an aqueous environment, open pores can fill with fluid, contributing to volumetric changes during thermal cycling. Indeed, the methacrylate‐based sealer EndoREZ showed no statistically significant differences in volume compared with the two other sealers, except for AH Plus at 1‐mm. EndoREZ also contains hydrophilic methacrylate resin like AH Plus, which may improve penetration into dentinal tubules, but also makes the sealer more responsive to environmental moisture (Donnelly et al. 2007). The present findings indicate that these changes were minimal, implying that they are unlikely to affect clinical outcomes.

The sealers MTA Fillapex and TotalFill BC displayed significantly smaller expansion than AH Plus. Their stability may be attributed to the surface formation of hydroxyapatite at the dentinal wall, enhanced under environmental conditions in vivo (Atmeh et al. 2012). Minimal sorption and a low coefficient of thermal expansion also contribute to the volumetric stability of calcium silicate cement‐containing sealers (Mert and Gençoğlu 2024; Camilleri 2007; Camilleri et al. 2013; FKG swiss endo 2025). Some publications (Mert and Gençoğlu 2024) have reported dimensional changes in TotalFill BC Sealer, but the present study indicates that MTA Fillapex and TotalFill BC sealers demonstrated less dimensional change than AH Plus in the apical region (Camilleri 2009).

MTA Fillapex is predominantly composed of salicylate resin, with an MTA content ranging from 13% to 15%. The moderately hydrophilic nature of the salicylate resin may contribute to water sorption, which in turn can promote expansion. At the same time, this hydrophilicity may also be associated with increased solubility, hydrolytic degradation of the polymer matrix, and subsequent microleakage.

However, when compared to AH Plus, MTA Fillapex has been reported to exhibit less dimensional change under artificial aging conditions (Camilleri 2009), which appears to be consistent with the present findings.

Less expansion was observed for the tricalcium silicate cement sealer, TotalFill BC. Calcium silicate cement setting requires water, and it gradually strengthens over 28 days, during which the capillary pores fill with water(Lim et al. 2020).

No statistically significant volumetric changes were observed in the total filling volume for any sealer after thermocycling. This outcome may be attributed to the insulating properties of dentin, which can act as a thermal barrier against temperature fluctuations, thereby reducing the transmission of temperature changes to the endodontic material.

Identical gutta‐percha cones (Reciproc R40) and one obturation technique were used in all experimental groups to reduce variability. Repeated thermal cycling induces expansion and contraction of gutta‐percha associated with phase transitions, potentially leading to interfacial gap formation. and thus can influence the overall stability of the obturation, especially in the apical region, where the changes were most pronounced (Liao et al. 2021). An important aspect of single‐cone obturation is the lack of chemical bonding between conventional gutta‐percha cones and most endodontic sealers. Conventional gutta‐percha is chemically inert and does not chemically bond to dentin or to sealers such as AH Plus, bioceramic sealers, or EndoREZ when used with standard gutta‐percha cones. Therefore, the long‐term stability of the obturation depends primarily on sealer dimensional stability, interfacial adaptation, penetration into dentinal irregularities, and the maintenance of a thin and homogeneous sealer layer (Al‐Hiyasat and Alfirjani 2019; Lim et al. 2020). As a result, thermocycling can lead to separation at the sealer–gutta‐percha interface, potentially compromising the integrity of the seal over time (Lee et al. 2002; Tay and Pashley 2007; Gale and Darvell 1999; Morresi et al. 2014). Thermocycling reproduces temperature fluctuations that may be experience interorally in the presence of moisture. Although materials placed within the root canal are exposed to less pronounced temperature changes than restorative materials, thermocycling remains a widely used and accepted method for generating dynamic stresses and assessing the long‐term behavior of dental materials (Morresi et al. 2014). While thermocycling cannot fully replicate clinical aging, it remains a widely accepted method for accelerated aging of dental materials. A limitation of this study is that aging was simulated exclusively by thermal cycling, without the inclusion of mechanical loading (e.g., fatigue). Future studies should combine thermal and mechanical aging protocols, with additional morphological and chemical analyses, to provide a more comprehensive understanding of the behavior of endodontic sealers under conditions that more closely resemble the in vivo environment over time.

## 5 Conclusion

Thermal cycling induced measurable volumetric changes in four endodontic sealers in teeth obturated with a single cone, particularly in the apical region of the canal. However, no statistically significant changes were observed in the total filling volume for any sealer after thermocycling. All materials exhibited both expansion and contraction across different regions, indicating a complex and material‐dependent response to thermal stress.

Among the tested sealers, AH Plus showed the greatest tendency toward expansion, whereas TotalFill BC Sealer and MTA Fillapex demonstrated smaller changes. These findings suggest that volumetric behavior is influenced by multiple underlying mechanisms, which may differ among materials and may have both beneficial and detrimental effects on sealing ability. Differences in the magnitude and nature of these changes may be relevant when considering long‐term sealing performance.

## Author Contributions

Petra Dijanic and Jurica Matijevic have contributed to the study's concept and design and to the acquisition of data. Ivan Tomasic has analyzed and interpreted the data. All authors (Petra Dijanic, Marko Katic, Ivan Tomasic, Ana Ivanisevic, and Jurica Matijevic) have performed the literature search and contributed to writing the manuscript. All authors have contributed to the manuscript substantially and have agreed to the final submitted version.

## Ethics Statement

The research was approved by the Ethics Committee of the School of Dental Medicine, University of Zagreb (Ethical approval No. 05‐PA‐15‐10/2017).

## Conflicts of Interest

The authors declare no conflicts of interest.

## Supporting information


Supporting File

## Data Availability

The data that support the findings of this study are available from the corresponding author upon reasonable request.
