# Supplementary figures and images for: Impact of Thermal Cycling on Volumetric Stability of Endodontic Filling Materials
Source: Clin Exp Dent Res. 2026 Jul 8;12(4):e70400. doi: 10.1002/cre2.70400 (PMC13345601; doi:10.1002/cre2.70400)

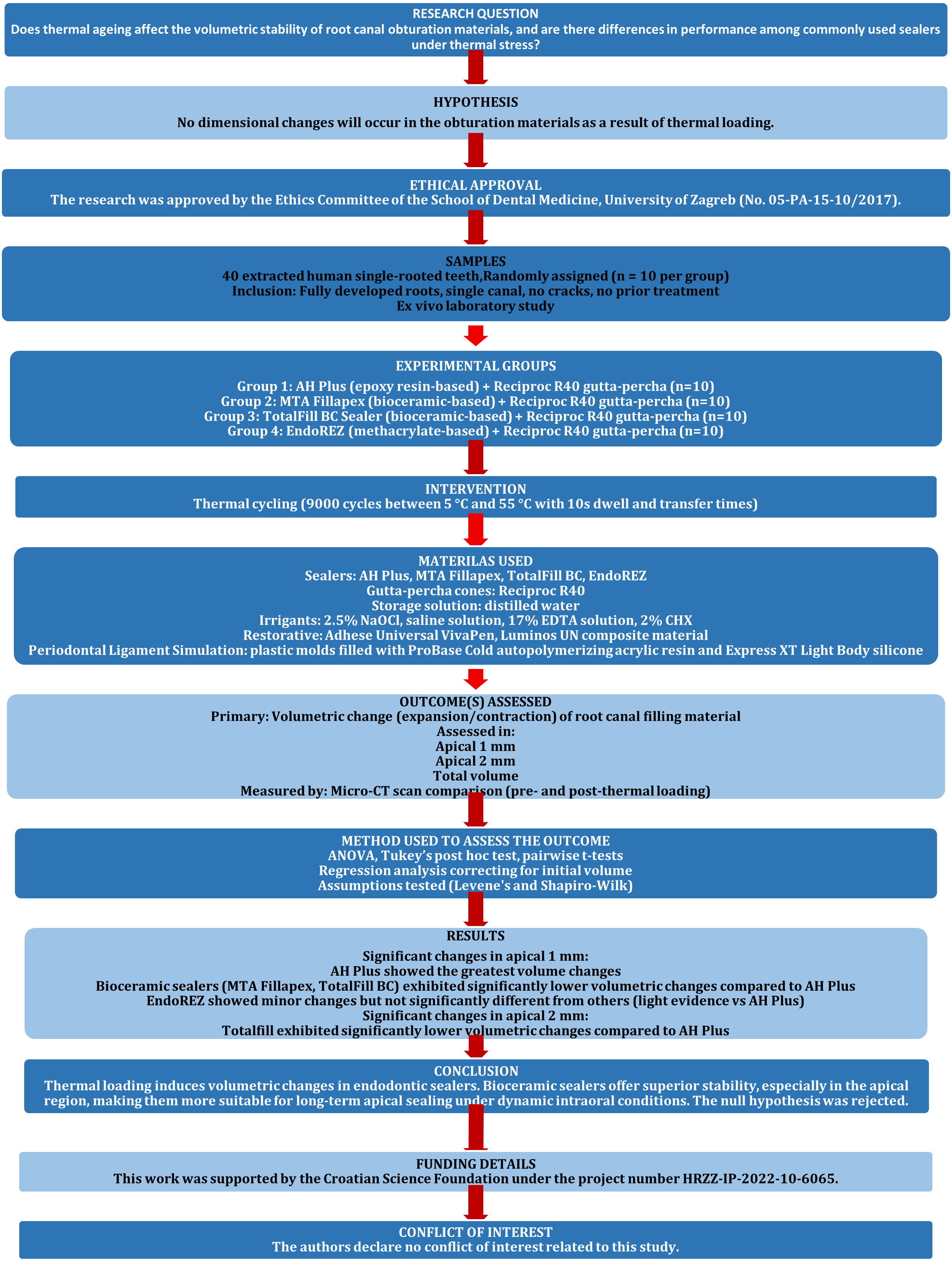


Figure S1. PRILE flowchart.

Supplement: Supplementary file 1 — Supporting File [file CRE2-12-e70400-s001.docx]
